# Analysis of gene expression changes during lipid droplet formation in HepG2 human liver cancer cells

**DOI:** 10.3892/mi.2024.131

**Published:** 2024-01-05

**Authors:** Mitsuru Chiba, Yuhei Ohsugi, Kana Matsumoto, Chisa Tayama

**Affiliations:** 1 Department of Bioscience and Laboratory Medicine, Graduate School of Health Sciences, Hirosaki University, Hirosaki, Aomori 036-8564, Japan; 2 Research Center for Biomedical Sciences, Hirosaki University, Hirosaki, Aomori 036-8564, Japan; 3 Department of Medical Technology, School of Health Sciences, Hirosaki University, Hirosaki, Aomori 036-8564, Japan

**Keywords:** oleic acid, lipid droplet, gene expression, microarray, HepG2, perilipin 2

## Abstract

Fatty liver is a condition of excessive triglyceride accumulation in hepatocytes. Additionally, hepatocytes exhibit a high degree of fat droplet accumulation during excessive alcohol consumption and metabolic syndrome. However, the molecular mechanisms involved in fat droplet formation remain unknown. The present study used an *in vitro* fatty liver formation model of the human liver cancer cell line, HepG2, to comprehensively search for fat droplet formation-related genes, and which exhibit changes in expression during fat droplet formation. Microarray analysis with extracted total RNA determined the genes that are involved in fat droplet formation and their expression was confirmed using quantitative polymerase chain reaction following the culture of the HepG2 cells in culture medium containing 0, 50, 200 and 500 µM of oleic acid for 24 h. The results revealed 142 genes demonstrating increased expression levels by >2.0-fold with oleic acid treatment and 426 genes demonstrating decreased expression levels. Perilipin 2 (*PLIN2*) was estimated as the gene most closely associated with fatty liver. Lipid droplet formation in the HepG2 cells induced by oleic acid led to the upregulation of *PLIN2* in a concentration-dependent manner. On the whole, the findings of the present study indicate the involvement of genes in oleic acid-induced lipid droplet formation in HepG2 cells; *PLIN2* in particular may play a crucial role in this process.

## Introduction

Fatty liver is a condition of excessive triglyceride accumulation inside hepatocytes. Known risk factors for fatty liver include excessive alcohol consumption, insulin resistance/lifestyle-related diseases, abnormal lipid metabolism and endocrine disorders (1,2). Among these, non-alcoholic fatty liver disease (NAFLD) refers to fatty liver due to excessive alcohol consumption. Of NAFLD cases, 80-90% of affected patients have NAFLD, while the remaining 10-20% have non-alcoholic steatohepatitis (NASH), which gradually deteriorates into fatty degeneration, inflammatory cell infiltration and balloon-like degeneration. If left untreated for a long period of time, the disease may quietly progress into fibrosis and may increase the risk of developing cirrhosis or hepatocellular carcinoma (3,4). Fat droplet accumulation in hepatocytes is one of the factors involved in both conditions, and understanding the mechanisms of fat droplet formation is critical for the prevention of fatty liver.

A model for intracellular fat droplet formation includes the method of treating 3T3-L1 mouse fibroblasts with insulin, isobutylmethylxanthine or dexamethasone to induce adipocyte differentiation (5). Treatment of the liver cancer cell line, HepG2, with oleic acid leads to the formation of intracellular fat droplets and can be used to study the mechanisms of fat droplet accumulation (6). These models are critical *in vitro* models for understanding the mechanisms of fat droplet formation.

In general, fat droplet formation in cells involve the endoplasmic reticulum. First, triglyceride synthase, which is present in the endoplasmic reticulum membrane in the cell, synthesizes triglycerides in the endoplasmic reticulum membrane and triglycerides accumulate to form the fat droplet lens (7). Gradually, the triglyceride lenses bud to the cytoplasmic side and separate from the endoplasmic reticulum membrane, causing fat droplet accumulation in the cytoplasm, which becomes fat droplets (7). One of the molecules associated with these fat droplet formations is Seipin, which contributes to their budding to the cytoplasmic side by preventing their budding to the lumenal side of the endoplasmic reticulum (8). However, fat droplet formation indicates the involvement of a number of other molecules. The present study used an *in vitro* fatty liver formation model of the liver cancer cell line, HepG2, to comprehensively search for fat droplet formation-related genes whose expression changes during fat droplet formation.

## Materials and methods

### Cells and cell culture

Adipogenesis was performed *in vitro* using the liver cancer cell line, HepG2. The Japanese Collection of Research Bioresources provided the HepG2 cells (JCRB1054; https://cellbank.nibiohn.go.jp/~cellbank/en/search_res_det.cgi?ID=2936), and Dulbecco's modified Eagle's medium (FUJIFILM Wako Pure Chemical Corporation) with 10% fetal bovine serum and antibiotics, including penicillin and streptomycin was used to culture the HepG2 cells. The cells were cultured at 37˚C in 5% CO_2_.

### Oleic acid solution

Bovine serum albumin (BSA) (FUJIFILM Wako Pure Chemical Corporation) was prepared by dissolving in 0.1 mol/l Tris-HCl (FUJIFILM Wako Pure Chemical Corporation) (pH 8.0) to a concentration of 5% and filtered through a 0.22-µm filter for sterilization. Oleic acid (FUJIFILM Wako Pure Chemical Corporation) was dissolved in 5% BSA solution to prepare 4 mM oleic acid solution. Oleic acid use solution was diluted to 0, 50, 200 and 500 µM with the aforementioned culture medium.

### Oil Red O staining solution

Oil Red O powder (FUJIFILM Wako Pure Chemical Corporation) was dissolved at 0.15 g with 50 ml isopropanol (FUJIFILM Wako Pure Chemical Corporation) to prepare the Oil Red O preservation solution. A total of 20 ml distilled water were added to 30 ml Oil Red O preservation solution, followed by incubation for 10 min, and filtering through a 0.22-µm filter. This filtrate was used as an Oil Red O staining solution.

### Oleic acid treatment and Oil Red O staining of HepG2 cells

The HepG2 cells were seeded at 5x10^3^ cells/well in eight-well chamber slides and cultured at 37˚C in 5% CO_2_. The culture medium containing 0, 50, 200 or 500 µM of oleic acid was used to replace the culture medium after 72 h followed by incubation at 37˚C in 5% CO_2_ for 24 h. Subsequently, the culture medium was discarded and the cells incubated with 4% paraformaldehyde (FUJIFILM Wako Pure Chemical Corporation) at room temperature for 30 min. Glass slides was washed three times with Dulbecco's phosphate-buffered saline (D-PBS) (-) (FUJIFILM Wako Pure Chemical Corporation). A drop of Oil Red O stain was added to each well followed by incubation at 60˚C for 10 min. The glass slides were then washed twice with D-PBS (-) and sealed with water-soluble sealant (Nichirei Biosciences). Cell counts and fat droplet areas in the images were analyzed using ImageJ version 1.52r (National Institutes of Health).

### Total RNA extraction

The HepG2 cells were seeded at 1x10^5^ cells/well in six-well plates and incubated at 37˚C in 5% CO_2_. The culture medium was replaced with 0, 50, 200 or 500 µM oleic acid after 72 h and the cells were incubated at 37˚C in 5% CO_2_ for 24 h. Total RNA was then extracted used the RNeasy Mini kit (Qiagen, Inc.) following the manufacturer's instructions. A NanoDrop spectrophotometer (NanoDrop Technologies; Thermo Fisher Scientific, Inc.) was used to assess the quality and concentration of total RNA. All RNA samples demonstrated 260/280-nm absorbance ratios of 1.8-2.0. An Agilent 2100 Bioanalyzer and an Agilent RNA 6000 Pico Kit (Agilent Technologies, Inc.) confirmed the peaks of total RNAs, following the manufacturer's instructions.

### Microarray analysis

In a previous study (9), the authors performed microarray analysis using 150 ng liver total RNA. Screening was performed for genes whose expression varied by >2.0-fold when treated with any oleic acid concentration compared to the untreated control. The obtained microarray data were registered with Gene Expression Omnibus (GSE248166). Furthermore, NetworkAnalyst (https://www.networkanalyst.ca/) was used to estimate the genes associated with fatty liver.

### Reverse transcription-quantitative polymerase chain reaction (RT-qPCR)

The expression levels of human perilipin (*PLIN*) family (*PLIN1*, *PLIN2*, *PLIN3*, *PLIN4* and *PLIN5*) and glyceraldehyde-3-phosphate dehydrogenase (*GAPDH*) mRNAs in HepG2 cells were examined using qPCR. cDNA was synthesized from 20 ng/µl total RNA using the Applied Biosystems^™^ High Capacity cDNA Reverse Transcription kit (Thermo Fisher Scientific, Inc.) according to the manufacturer's instructions. qPCR was performed using a FastStart Universal SYBR-Green Master (MilliporeSigma), 10 µM forward and reverse primer pairs (Tables I and S1), and the StepOne Plus Real-time PCR system (Thermo Fisher Scientific, Inc.) under the following conditions: 10 min at 95˚C, followed by 40 cycles each of 95˚C for 15 sec, and 60˚C for 60 sec. *GAPDH* was used as an internal control. The quantification of gene expression was calculated using the 2^-ΔΔCq^ method based on previous reports (10-12).

### Statistical analysis

All statistical analyses were performed using Statcel 3 software (OMS Publishing Inc.). A one-way analysis of variance was performed followed by Tukey-Kramer post hoc analysis to compare the results of the three groups. P*-*values <0.05 were considered to indicate statistically significant differences.

## Results

### Fat droplet formation induced by treatment with oleic acid in HepG2 cells

**Figure 1:**
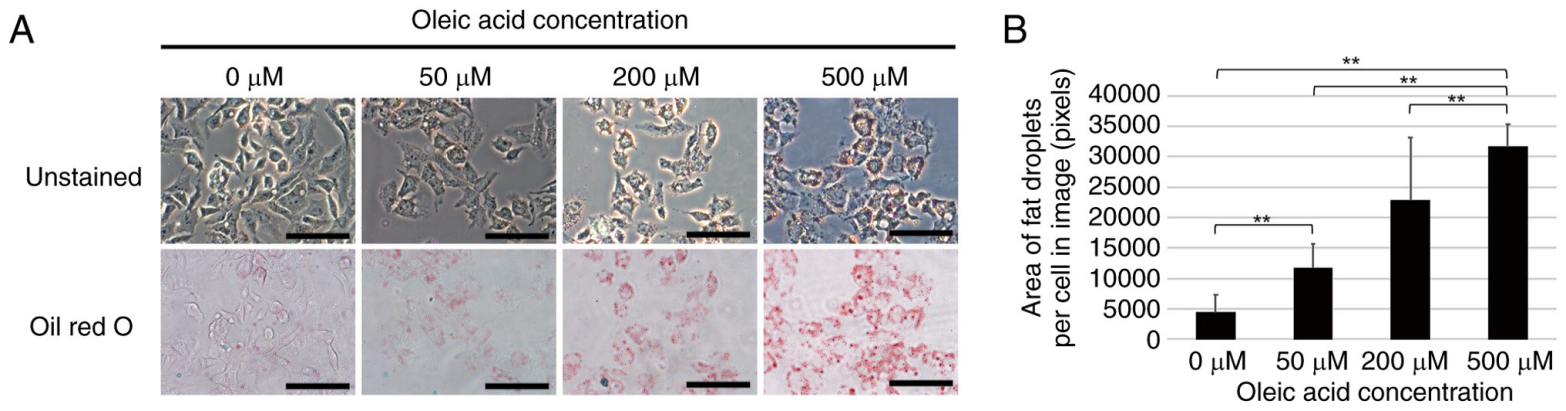
Fat droplet formation induced by oleic acid treatment in HepG2 cells. (A) Oil Red O staining to indicate fat droplet formation in HepG2 cells. Oleic acid at final concentrations of 0, 50, 200, or 500 µM was added to the culture medium of HepG2 cells. Oil Red O staining was performed after 24 h. Images were obtained with a 20X objective lens. Scale bars, 100 µm. (B) Area of fat droplets per cell shown in the images in panel A. Cell counts and fat droplet areas in the images were analyzed using ImageJ software. Values are presented as the mean ± 2 standard deviation (SD) (n=5) ^**^P<0.01.

A model of fat droplet formation was established by treatment of the HepG2 cells with oleic acid in an aim to elucidate the mechanisms of fat droplet formation in NAFLD. Oleic acid was added at final concentrations of 0, 50, 200 or 500 µM to the culture medium of HepG2 cells and Oil Red O staining was performed following 24 h of culture. The results revealed a concentration-dependent increase in the number of fat droplets in the cells treated with oleic acid (Fig. 1).

### Changes in gene expression during fat droplet formation induced by oleic acid treatment

**Figure 2:**
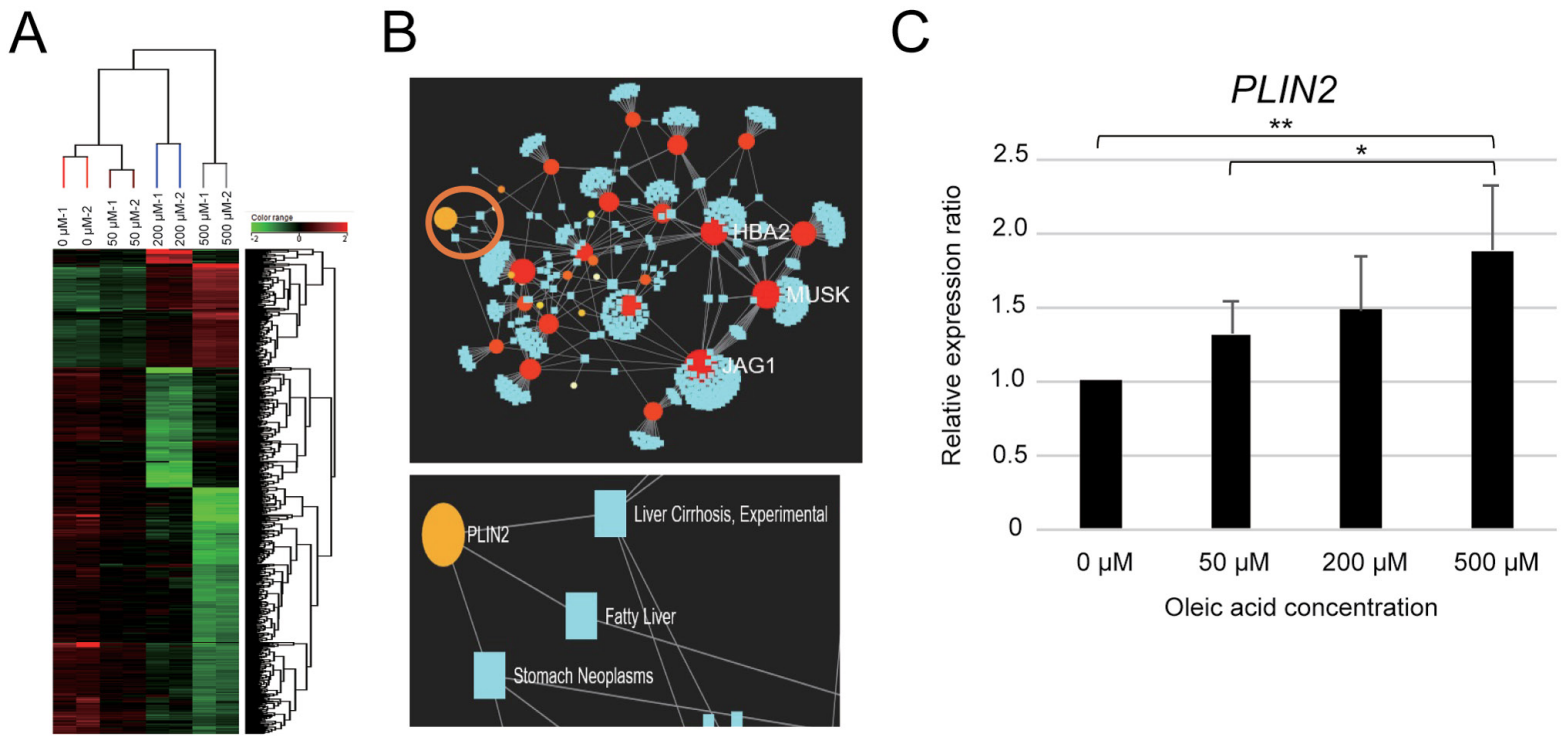
Changes in gene expression during fat droplet formation induced by oleic acid treatment. (A) Cluster analysis of expression genes exhibiting changes in expression in oleic acid-treated HepG2 cells. Genes with at least a ≥2.0-fold change in expression in at least one of the 50, 200 and 500 µM treatments compared to the 0 µM treatment were selected. (B) Association between expression variation genes and diseases by NetworkAnalyst. The analysis used genes whose expression was upregulated by >2.0-fold in at least one of the 50, 200, or 500 µM treatments compared to the 0 µM treatment. Light blue squares indicate disease, red circles indicate genes and orange circles indicate liver function-related genes. (C) *PLIN2* expression analysis in oleic acid-treated HepG2 cells. Values are presented as the mean ± 2 SD (n=3). ^*^P<0.05 and ^**^P<0.01. *PLIN2*, perilipin-2.

A microarray analysis was performed on the HepG2 cells 24 h following treatment with the respective oleic acid concentrations to determine the changes in gene expression that occur during lipid droplet formation. The genes whose expression increased by >2.0-fold in the cells treated with 50, 200 or 500 µM compared to 0 µM oleic acid accounted for 142, while the number of genes whose expression decreased was 426 (Fig. 2A). These results revealed that oleic acid treatment induced various changes in gene expression in HepG2 cells.

NetworkAnalyst was used to estimate and determine the genes associated with NAFLD among the genes which exhibited a variable expression in the oleic acid-treated HepG2 cells. The *PLIN2* gene was found to be associated with ‘fatty liver’ and ‘liver cirrhosis’ (Fig. 2B). Additionally, *PLIN2* expression in the HepG2 cells exhibited a concentration-dependent increase following treatment with oleic acid (Fig. 2C). However, there was no statistically significant difference in the levels of *PLIN1*, *PLIN3* and *PLIN4* in the HepG2 cells treated with oleic acid. Of note, *PLIN5* expression exhibited a significant decrease in the cells treated with oleic acid (Fig. S1). These results indicate the association between *PLIN2* and the development of NAFLD.

## Discussion

NASH is a condition of NAFLD characterized by fatty degeneration, inflammatory cell infiltration and balloon-like degeneration (13); however, the mechanisms responsible for fat droplet formation in fatty degeneration remain unknown. Therefore, the present study used an experimental cell model of fat droplet formation by using HepG2 liver cancer cells treated with oleic acid. Oleic acid is a *cis*-unsaturated fatty acid and is used as a model for fat droplet formation as it is less toxic and more sensitive to cholesterol acyltransferases and diacylglycerol acyltransferases that are involved in fat droplet formation than other saturated and trans fatty acids (14,15). In the present study, treatment of the HepG2 cells with oleic acid induced a concentration-dependent increase in the number of fat droplets (Fig. 1). It was also revealed various gene expression changes that occurred in HepG2 cells treated with various concentrations of oleic acid in an aim to identify genes involved in fat droplet formation. Network analysis identified *PLIN2* as a gene associated with fatty liver and cirrhosis (Fig. 2B), and its expression increased with the increasing number of fat droplets (Fig. 2C). These results indicate that *PLIN2* plays a critical role in fat droplet formation.

*PLIN2* is one of the five *PLIN* family members; it exists as a protein that binds to fat droplet surfaces, and it plays a role in stabilizing fat droplets by interfering with the breakdown of triglycerides by enzymes (16,17). A previous study examining the distribution of the *PLIN* family in the body revealed that all *PLIN* family members were expressed in the majority of organs, and were particularly overexpressed in adipocytes and mammary glands, as well as in the liver (18). The present study also examined the expression of not only the *PLIN2* gene, but also that of the *PLIN1*, *PLIN3*, *PLIN4*, and *PLIN5* genes during lipid droplet formation using RT-qPCR, and found that only *PLIN2* expression was increased in a concentration-dependent manner following oleic acid treatment in this model of fat droplet formation (Figs. 1 and S1). On the other hand, *PLIN5* expression was significantly downregulated by oleic acid treatment. This suggests that *PLIN2* plays a crucial role in lipid droplet formation induced by oleate treatment and *PLIN5* plays an antagonistic role. To the best of our knowledge, to date, no previous studies have focused on changes in *PLIN* family expression in a model of fat droplet formation induced by the oleic acid treatment of HepG2 cells, and the present study is the first to demonstrate that *PLIN2* and *PLIN5* exhibit opposite expression patterns. Recently, Jin *et al* (19) demonstrated that the overexpression of *PLIN2* in HepG2 cells decreased *PLIN5* expression, while the knockdown of *PLIN2* increased *PLIN5* expression. This suggests that an increased *PLIN2* expression in HepG2 cells may be associated with a decreased *PLIN5* expression. However, the functional association between *PLIN2* and *PLIN5* in fat droplet formation remains unclear and warrants further investigation. In addition, microarray analysis was performed on a small number of samples in the present study, and statistical analysis was not sufficient. Although changes in the expression of *PLIN2* and other *PLIN* family members could be reproduced by RT-qPCR, it is necessary to confirm the expression of other genes individually using RT-qPCR or other methods.

Several studies have reported the association between *PLIN2* and liver diseases, and its involvement in fatty liver has been reported in *in vivo* analysis, since *PLIN2* is closely related to fat droplet accumulation in the liver. Nocetti *et al* (20) reported that hepatocytes from mice with NAFLD induced by a high-fat diet exhibited an increase in *Plin2* expression along with highly oxidized fat droplets. Griffin *et al* (21) also demonstrated that diet-induced hepatic lipidosis was ameliorated in liver-specific *Plin2* knockout mice. This suggests that *Plin2* is associated with NASH/NAFLD. On the other hand, Mak *et al* (22) demonstrated that alcohol consumption in rats induced a renewal of *Plin2* expression. Carr *et al* (23) indicated that alcohol consumption in *Plin2* knockout mice suppressed the development of fatty liver. These reports indicate that *PLIN2* contributes to the development of fatty liver by increasing *PLIN2* expression in the liver, regardless of alcohol intake. Notably, there is a single nucleotide polymorphism in human *PLIN2*, and Faulkner *et al* (24) reported that humans carrying the rs35568725 mutant allele encoding Ser251Pro are at an increased risk of developing NASH. Recently, *PLIN2* was highlighted as a potential therapeutic target for NASH/NAFLD (25). In the future, it is hoped that the pharmacological suppression of *PLIN2* will lead to the development of novel therapies which can be used combat fatty liver disease.

## Supplementary Material

Changes in *PLIN1*, *PLIN3*, *PLIN4* and *PLIN5* expression during fat droplet formation induced by oleic acid treatment. (A-D) *PLIN1*, *PLIN3*, *PLIN4* and *PLIN5* expression analysis in oleic acid-treated HepG2 cells. Values are presented as the mean ± 2 SD (n=3). ^*^P<0.05. *PLIN*, perilipin.

Primer pairs used for reverse transcription quantitative PCR.

## Figures and Tables

**Table I:** Primer pairs used for reverse transcription-quantitative PCR.

Primer	Sequence (5' to 3')	Amplicon size (bp)
*PLIN2* forward	TCAGCTCCATTCTACTGTTCACC	74
*PLIN2* reverse	CCTGAATTTTCTGATTGGCACT	
*GAPDH* forward	AGCCACATCGCTCAGACAC	66
*GAPDH* reverse	GCCCAATACGACCAAATCC	

*PLIN2*, perilipin-2.

## Data Availability

The datasets used and/or analyzed during the present study are available from the corresponding author upon reasonable request. The obtained microarray data were registered with Gene Expression Omnibus (GSE248166).
