# Supplementary material for: Analysis of gene expression changes during lipid droplet formation in HepG2 human liver cancer cells
Source: Med Int (Lond). 2024 Jan 5;4(1):7. doi: 10.3892/mi.2024.131 (PMC10811445; doi:10.3892/mi.2024.131)

Figure S1. Changes in *PLIN1*, *PLIN3*, *PLIN4* and *PLIN5* expression during fat droplet formation induced by oleic acid treatment. (A-D) *PLIN1*, *PLIN3*, *PLIN4* and *PLIN5* expression analysis in oleic acid-treated HepG2 cells. Values are presented as the mean ± 2 SD (n=3). *P<0.05. *PLIN*, perilipin.

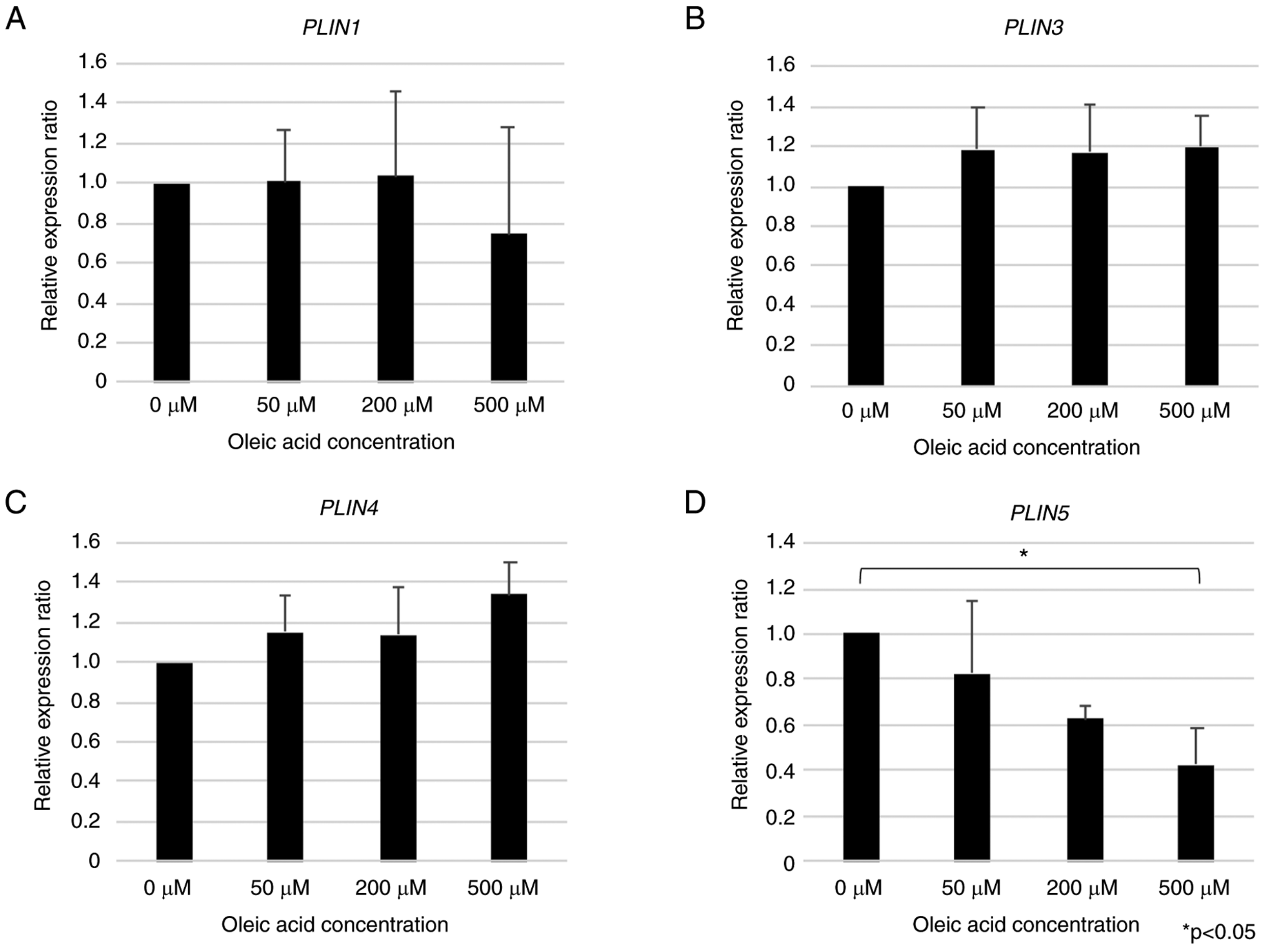

Supplement: Changes in PLIN1, PLIN3, PLIN4 and PLIN5 expression during fat droplet formation induced by oleic acid treatment. (A-D) PLIN1, PLIN3, PLIN4 and PLIN5 expression analysis in oleic acid-treated HepG2 cells. Values are presented as the mean ± 2 SD (n=3). *P<0.05. PLIN, perilipin. [file Supplementary_Data1.pdf]
