# Supplementary material for: Analysis of gene expression changes during lipid droplet formation in HepG2 human liver cancer cells
Source: Med Int (Lond). 2024 Jan 5;4(1):7. doi: 10.3892/mi.2024.131 (PMC10811445; doi:10.3892/mi.2024.131)
Supplement: Primer pairs used for reverse transcription quantitative PCR. [file Supplementary_Data2.pdf]

Table S1. Primer pairs used for reverse transcription-quantitative PCR.

Primer	Sequence (5’ to 3’)	Amplicon size (bp)
PLIN1 forward	TGAACATTAAAGGGAAGAAGTTGAA	96
PLIN1 reverse	TTCTCCTGCTCAGGGAGGT	
PLIN3 forward	AAGCAGAGGGCACAGGAG	74
PLIN3 reverse	ACGCCTTGCTTGACAGTTTC	
PLIN4 forward	AGTTCCAAGCCAGGGACAC	68
PLIN4 reverse	CTGCTGGGCCTTTTCAATC	
*PLIN5*forward	TTCGGAGACGGTGGTGAC	64
*PLIN5*reverse	GTCCACCACACCCGTGAC	

PLIN, perilipin.
